# Data-driven encoding for quantitative genetic trait prediction

**DOI:** 10.1186/1471-2105-16-S1-S10

**Published:** 2015-02-18

**Authors:** Dan He, Zhanyong Wang, Laxmi Parida

**Affiliations:** 1 IBM T.J. Watson Research, Yorktown Heights, NY, USA; 2 Department of Computer Science, University of California, Los Angeles, USA

**Keywords:** Quantitative Genetic Trait Prediction, Data-driven Encoding, Epistasis, Ridge Regression

## Abstract

**Motivation:**

Given a set of biallelic molecular markers, such as SNPs, with genotype values on a collection of plant, animal or human samples, the goal of quantitative genetic trait prediction is to predict the quantitative trait values by simultaneously modeling all marker effects. Quantitative genetic trait prediction is usually represented as linear regression models which require quantitative encodings for the genotypes: the three distinct genotype values, corresponding to one heterozygous and two homozygous alleles, are usually coded as integers, and manipulated algebraically in the model. Further, epistasis between multiple markers is modeled as multiplication between the markers: it is unclear that the regression model continues to be effective under this. In this work we investigate the effects of encodings to the quantitative genetic trait prediction problem.

**Results:**

We first showed that different encodings lead to different prediction accuracies, in many test cases. We then proposed a data-driven encoding strategy, where we encode the genotypes according to their distribution in the phenotypes and we allow each marker to have different encodings. We show in our experiments that this encoding strategy is able to improve the performance of the genetic trait prediction method and it is more helpful for the oligogenic traits, whose values rely on a relatively small set of markers. To the best of our knowledge, this is the first paper that discusses the effects of encodings to the genetic trait prediction problem.

## Background

Whole genome prediction of complex phenotypic traits using high-density genotyping arrays has attracted a lot of attention, as it is relevant to the fields of plant and animal breeding and genetic epidemiology [1-8]. Given a set of biallelic molecular markers, such as SNPs, with genotype values encoded as {0, 1, 2} on a collection of plant, animal or human samples, the goal is to predict the quantitative trait values by simultaneously modeling all marker effects.

One of the earliest, though still very relevant, treatments of genomic selection was given in [1]. In the article, the authors present four approaches: *least-squares estimation*, *BayesA*, *BayesB*, and *rrBLUP *(Ridge-Regression BLUP), based on a linear model of the marker effects on the trait being studied. The latter three methods are still competitive with the state-of-art techniques, and have also spawned a number of interesting variants. Specifically, rrBLUP [1,9] has been used widely for trait prediction where it builds a linear model by fitting all the genotypes, and the coefficient computed for each marker can be considered as a measure of the importance of the marker. The name rrBLUP stands for "ridge-regression" BLUP, where BLUP stands for the standard "best linear unbiased prediction" approach used in the field. rrBLUP can be viewed as either ridge-regression with a specific shrinkage parameter, or a particular mixed model equation with certain variance components [10,11]. The rrBLUP method has the benefits of the underlying hypothesis of normal distribution of the trait value and the marker effects, which is well suited for highly polygenic traits; rrBLUP is computationally efficient and robust, which makes it one of the most commonly used models in whole genome prediction. Other popular predictive models are Elastic-Net, Lasso, Ridge Regression [12,13], Bayes A, Bayes B [1], Bayes *C_π _*[14], and Bayesian Lasso [15,16], as well as other machine learning methods.

The genetic trait prediction problem is defined as follows. Given *n *training samples, each with *m *≫ *n *genotype values (we use "feature", "marker", "genotype", "SNP" interchangeably) and a trait value, and a set of *n′ *test samples each with the same set of genotype values but without trait value, the task is to train a predictive model from the training samples to predict the trait value, or phenotype of each test sample based on their genotype values. Let *Y *be the trait value of the training samples. The problem is usually represented as the following linear regression model:

(1) $$Y=\beta_{0}+\overset{m}{\underset{i=1}{\sum }}\beta_{i}X_{i}+e_{l}$$

where *X_i _*is the *i*-th genotype value, *m *is the total number of genotypes and *β_i _*is the regression coefficient for the *i*-th genotype, *e_l _*is the error term. We call this model *single marker model*.

The above model assumes that only the single markers, or main effects, play a role for the prediction. However, it is known that the interactions of the genotypes may also contribute to the genetic traits under certain conditions, which is known as *Epistasis *[17]. The pairwise epistasis between two genotypes *X_i _*and *X_j _*is often modeled as the product of the two genotype values. Therefore, with the traditional representation, the linear regression model with pairwise epistasis effects is modified as the following:

(2) $$Y=\beta_{0}+\overset{m}{\underset{i=1}{\sum }}\beta_{i}X_{i}+\overset{m}{\underset{i,j}{\sum }}\alpha_{i,j}X_{i}X_{j}+e_{l}$$

where *X_i_X_j _*is the product of the genotype values of the *i*-th and *j*-th genotype and it denotes the interaction of the two genotypes, *α_i,j _*represents the coefficient for the interaction. Thus in this epistasis model, the epistasis effects are considered as augmented genotypes besides the original genotype matrix *X*. We call this model *epistasis model*.

Genotypes for a marker can be either homozygous or heterozygous. For Genome Wide Association Study (GWAS), we only need to identify the association between a marker and the case/control trait. Therefore, we care more about whether genotypes are homozygous or heterozygous and the frequency of the alleles. They don't necessarily need to be quantitative. They are usually represented as a pair of alleles, for example "AA" and "TT" for homozygous genotypes, "AT" for heterozygous genotype.

On the other hand, for genetic trait prediction problem, in Equation 1 and 2, the genetic trait values *Y *are quantitative. Thus the genotypes *X_i _*need to be quantitative as well. Researchers generally assign three distinct encodings to the three possible genotype values. A few common sets of encodings for genotypes are {0, 1, 2}, where 0 and 2 are for homozygous genotypes and 1 is for heterozygous genotype, and {−1, 0, 1}, where -1 and 1 are for homozygous genotypes and 0 is for heterozygous genotype.

As genotypes need to be encoded, different encodings may lead to different prediction accuracies, especially for the epistasis model. This is because the multiplications of different encodings are different. For example, the multiplication of two heterozygous genotypes for encoding {0, 1, 2} is 1 × 1 = 1, but for encoding {−1, 0, 1} is 0 × 0 = 0. It's not clear which encoding should we use nor which encoding will produce better results. Another unreasonable setting is different interactions of the genotypes may have the same value. For example, the multiplication of two genotypes 0, 1 and 0, 2 are both 0. But there is no biological interpretation why the two genotype interactions contribute "identically" to the trait.

Based on the above observations, we developed a novel data-driven encoding method where the encoding of the genotypes depends on the data itself. The basic idea is straitforward: For each genotype *g *of each marker *i*, we identify the set of trait values for the samples whose genotype is *g *at marker *i*. Then we take the average of this set of traits and replace the genotype with this average value. Thus all the genotypes can be determined by the data itself and the encoding allows each marker to be encoded differently. We call this encoding method *pure data-driven encoding*.

In the traditional encoding, heterozygous genotype is the average value of the two homozygous genotypes, thus from the encoding we could tell which one is heterozygous genotype, which ones are homozygous genotypes. One problem of the pure data-driven encoding is that we can not distinguish between the homozygous genotype and heterozygous genotype any more, as their encodings completely depend on the data. So we propose a second version of the encoding, where we compute the new encoding for the two homozygous genotypes the same as in the pure data-driven encoding method, but we compute the new encoding for the heterozygous genotype as the mean of the whole data set. We call this encoding method *hybrid data-driven encoding*. More details will be given in the methods section.

## Related work

Lots of techniques have been applied to the genetic trait prediction problem defined in Equation 1. Consider the typical situation for linear regression, where we have the training set $y\in {\mathbb{R}}^{l}$, $x\in {\mathbb{R}}^{l\times n}$, in a standard linear regression, we wish to find parameters *β*_0_, *β *such that the sum of square residuals, $\sum_{i=1}^{l}{\left(y_{i}-\beta_{0}-x_{i}^{⊤}\beta \right)}^{2}$, is minimized.

The *lasso *approach [12,13] uses an additional *l*_1 _penalty which aims to achieve a sparse solution. This idea has even been extended to *group lasso *where variable are included or excluded in groups [18,19]. Alternatively *Ridge regression *(or *Tikhonov regularization*)[20] uses an *l*_2 _penalty which is ideal for the case when many predictors have non-zero coefficients. *Elastic-net *uses both an *l*_1 _and *l*_2 _penalty with a trade off parameter between the two [21]. Consequently lasso and ridge regression can be seen as special cases of Elastic-net. See [22] and references therein. The Elastic-net problem can be stated as

(3) $$\begin{array}{c} \underset{\left(\beta_{0},\beta \right)\in {\mathbb{R}}^{n+1}}{min}\left[\frac{1}{2l}\overset{l}{\underset{i=1}{\sum }}{\left(y_{i}-\beta_{0}-x_{i}^{⊤},\cdot \beta \right)}^{2}+\lambda P_{\alpha }\left(\beta \right)\right],\\ \;\text{where}\\ P_{\alpha }\left(\beta \right)=\left(1-\alpha \right)\frac{1}{2}||\beta |{|}_{l_{2}}^{2}+\alpha ||\beta |{|}_{l_{1}}. \end{array}$$

Thus when *α *= 1 corresponds to lasso and *α *= 0 corresponds to ridge.

Elastic-Net (with non-zero *α*) can be easily extended for genome wide associate studies by use of the non-zero *β *parameters selected when training the data. That is, the *l*_1 _penalty achieves a sparse solution, and in turn signals which variables contribute most when training on the data.

*rrBLUP (Ridge regression BLUP) *[1,9] is one of the most popular methods for genetic trait prediction. rrBLUP simply is ridge regression with a specific choice of *λ *in (3). Specifically, Meuwissen et al. [23] assumes that the *β *coefficients are iid from a normal distribution such that *β_i _*~ *N *(0, *σ_β_*). Then the choice of $\lambda =\sigma_{e}^{2}/\sigma_{\beta }^{2}$ where $\sigma_{e}^{2}$ is the residual error. In this case, the ridge regression penalized estimator is equivalent to best linear unbiased predictor (BLUP) [24].

Support vector machines (SVMs) are a tool in statistics and machine learning for the task of supervised learning [25-29] used for either classification or regression. Here we are interested in the latter case. Following [30], given a training set (**x***_i_, y_i_*), *i *= 1,...*l*, where $x_{i}\in {\mathbb{R}}^{n}$, the goal of *ε*-SV regression is to find a function *f*(**x**) that is at most *ε *deviation from the training data *y_i _*over the training data **x***_i_*, while remaining as flat as possible in the feature space. Training an SVM requires solving

(4) $$\begin{array}{ll} \;\underset{w,b,\xi }{\min} & \frac{1}{2}w^{⊤}w+C\overset{l}{\underset{i=1}{\sum^{\text{​}}}}\xi_{i}\\ \text{subject to} & y_{i}(w^{⊤}\phi (x_{i})+b)\geq 1-\xi_{i}-\varepsilon ,\\ \;\xi_{i}\geq 0. & \end{array}$$

The data vectors **x***_i _*are mapped to another space via the function *ϕ*, and SVM attempts to fit the data in this higher dimensional space. Thus, the choice of *ϕ*, referred to as the *kernel*, has a large impact. Four kernels are usually used:

$$\begin{array}{ll} \text{ Linear}: & u^{⊤}v,\\ \text{Polynomial}: & {\left(\gamma u^{⊤}v+r\right)}^{d},\gamma >0,\\ \text{ Radial}:\; & \exp(-\gamma ||u-v|{|}^{2}),\gamma >0,\\ \text{ Sigmoid}: & \mathrm{tann}(\gamma u^{⊤}v+r). \end{array}$$

Support vector regression involves solving Equation 4 given training data. The vector **w**, the choice of the kernel, and the choice of kernel parameters, used previously to solve Equation 4 gives a model capable of predicting future data.

The above work all aim to solve single marker genetic trait prediction. There are also lots of existing work on epistasis models for GWAS. As exhaustive search of all possible epistasis interactions is infeasible even for a small number of markers, greedy strategies [31-36] have been applied to detect epistasis effects where a subset of high-marginal effect markers, which are markers that contribute to the trait themselves, are first selected. Then the test is conducted either between all the markers in this subset or between the markers in this subset and the remaining markers. These strategies, however, miss all the possible epistasis between the low-marginal effect markers, which are shown to exist [17]. Xiang et al. [37] proposed an optimal algorithm to efficiently detect epistasis without conducting an extensive search. A data structure is created to effectively prune interactions that are potentially insignificant. These work focus on GWAS and they do not require a quantitative encoding. As a result, none of the existing work investigated the effects of encoding for genetic trait prediction, where quantitative encoding is a must. As multiplication is one of the most popular epistasis models, in this work, we consider only the multiplication model for epistasis.

## Methods

Genotype usually has three values, one for the homozygous major allele, one for the homozygous miner allele and one for the heterozygous allele. In the traditional encoding {0, 1, 2}, 1 is the value for the heterozygous genotype, 0 and 2 are for the homozygous genotype, one on major allele, one on miner allele. All the markers are encoded the same way. In this work, we propose two data-driven encoding strategies. The first encoding strategy is called *pure data-driven encoding*. The new encoding for genotype of value 0 at marker *i *is computed as *E*(*i*, 0) = *Ave*(*trait*(*i*, 0)), where *E*(*i*, 0) is the new encoding for genotype of value 0 at maker *i*, *trait*(*i*, 0) is the set of traits for the samples whose genotypes are 0 at marker *i*, *Ave*() is the function to compute the average value. Thus we also have *E*(*i*, 1) = *Ave*(*trait*(*i*, 1)), *E*(*i*, 2) = *Ave*(*trait*(*i*, 2)).

The first encoding strategy is pure data-driven. It doesn't distinguish homozygous genotypes with heterozygous genotypes. Thus we developed the second encoding strategy *hybrid data-driven encoding*, where we still have *E*(*i*, 0) = *Ave*(*trait*(*i*, 0)) and *E*(*i*, 2) = *Ave*(*trait*(*i*, 2)) for the two homozygous genotypes 0, 2. However, for the heterozygous genotype 1, we have *E*(*i*, 1) = *Ave*(*trait*(*i*, {0, 1, 2})), where *trait*(*i*, {0, 1, 2}) indicates the trait values for all the samples. The intuition of this encoding strategy is from the traditional encoding that the encoded value of the heterozygous genotype should be the average of the values for homozygous genotypes. However, we would also like to take the trait values into consideration so that the encoding of the heterozygous genotype not only depends on the encoding of the homozygous genotypes, but also the corresponding trait values. As we will show later in the experiments, the pure data-driven encoding is in general worse than the traditional encoding, and the hybrid data-driven encoding is in general better than the traditional encoding.

**Figure 1:**
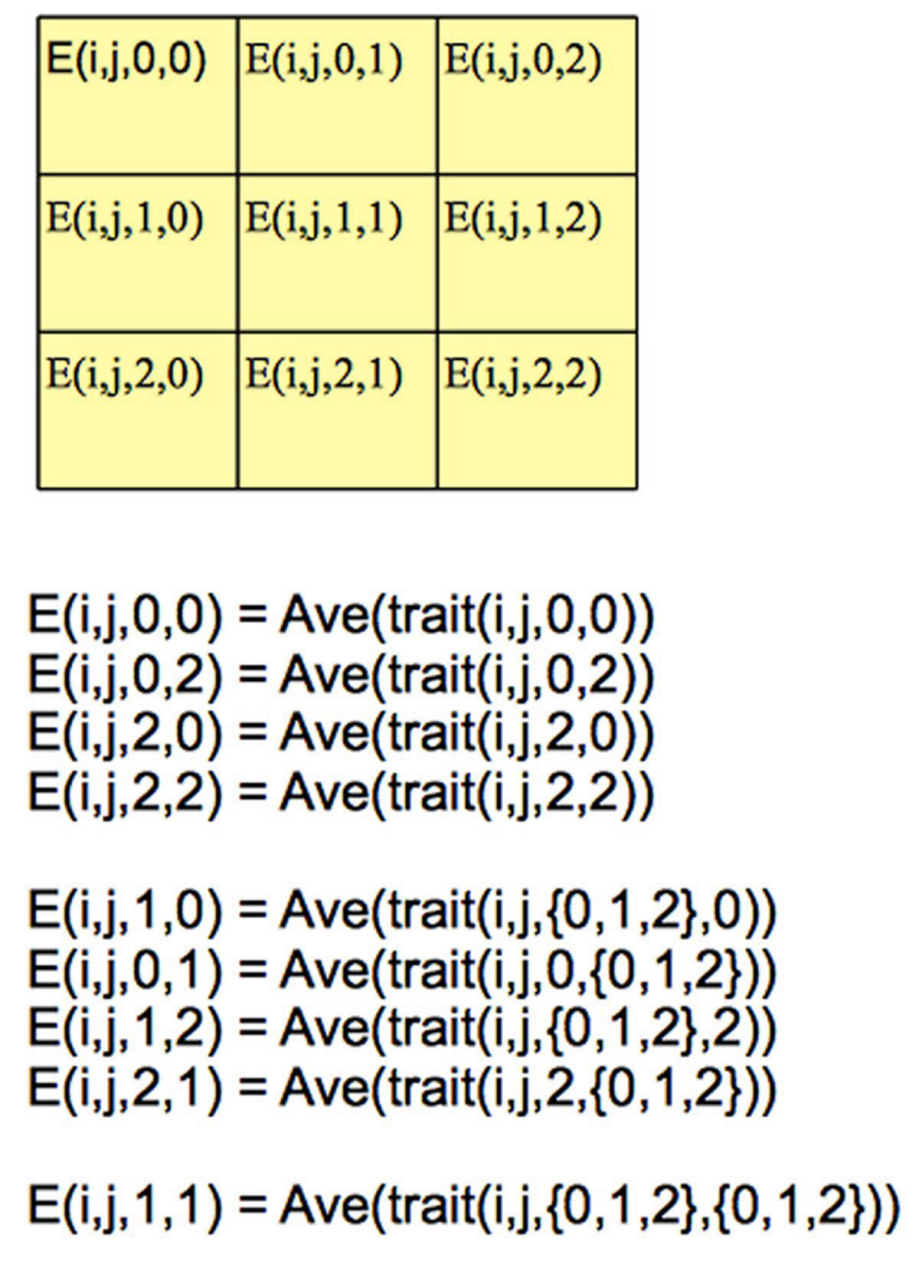
**The data-driven encoding for pairwise epistasis of markers *i, j***.

The data-driven encoding strategies can be naturally extended to pairwise epistasis effects or even higher dimensional epistasis effects. As the hybrid data-driven encoding has better performance, the extension is based on the hybrid data-driven encoding method. As shown in Figure 1, for pairwise epistasis effects, given the traditional encoding {0, 1, 2}, we have 9 possible combinations for markers *i *and *j*, organized in the 3 × 3 grid matrix. Assuming 0 is the traditional encoding for homozygous genotype with major allele, 2 is the traditional encoding for homozygous genotype with miner allele, 1 is traditional encoding for heterozygous genotype, then the cell (0, 0) (from now on, for simplicity, we ignore the marker indices *i, j *for the cell) is the traditional encoding for a pair of homozygous genotypes, both with major allele, the cell (2, 2) is the traditional encoding for a pair of homozygous genotypes, both with miner allele, the cell (1, 2) is the traditional encoding for a pair of heterozygous genotype and homozygous genotype with miner allele. The meaning of the other cells can be inferred similarly.

Our goal is to encode each cell using the data-driven approach. We first compute the data-driven encoding for the four corner cells (0, 0), (0, 2), (2, 0), (2, 2) as the average of their corresponding trait values, as shown in Figure 1. For example, *E*(*i, j*, 0, 0) = *Ave*(*trait*(*i, j*, 0, 0)), where *trait*(*i, j*, 0, 0) is the set of traits for the samples whose traditional genotypes at marker *i, j *are 0 and 0, respectively. Then for the cells (1, 0), (0, 1), (2, 1), (1, 2), we compute their data-driven encoding by extending the encoding strategy for single markers. For example, *E*(*i, j*, 1, 0) = *Ave*(*trait*(*i, j*, {0, 1, 2}, 0)), where *trait*(*i, j*, {0, 1, 2}, 0) is the set of traits for the samples whose traditional genotype at marker *i *is 0 or 1 or 2, and at marker *j *is 0, respectively. The intuition is that we consider the encoding for the three cells (0, 0), (1, 0), (2, 0) for the marker pair *i, j *as fixing the genotypes for marker *j *as 0. Then the problem is converted to computing the encoding for a single marker *i*, whose genotype can be either 0, or 1, or 2. Similar encoding strategies are also applied on the cells [(0, 2), (1, 2), (2, 2)], [(0, 0), (0, 1), (0, 2)], [(2, 0), (2, 1), (2, 2)] to compute the encodings for cells (1, 2), (0, 1), (2, 1), respectively.

Finally for the cell in the center (1, 1), we compute its data-driven encoding as the average of all the traits, namely *E*(*i, j*, 1, 1) = *Ave*(*trait*(*i, j*, {0, 1, 2}, {0, 1, 2})). This is again a strait-forward extension of the encoding strategy for single markers.

The same data-driven encoding algorithm can be further extended to higher dimensional epistasis effects. In this work, we only focused on the application of the data-driven encoding algorithm on single marker and pairwise epistasis effects.

As we will show later in the experiments in Section, the data-driven encoding not only is able to improve the performance for the epistasis model, but also for the single marker model. Next we investigate the reason that the data-driven encoding is able to improve the performance of the prediction in general. For the traditional encoding, the same genotype of different markers are encoded with the same value. However, from GWAS, we know that different SNPs have different associations to the trait. Thus the markers contribute differently to the trait. Therefore, constraining the same genotype of different markers with the same value may not be appropriate to obtain the best contrast factors, or the coefficients of the markers for the regression. Our encoding method, on the contrary, allows the same genotype of different markers to have different values. And the higher the association between the marker and the trait is, the more close the new encoding values to the trait value. The regression based on the new encodings thus can more effectively identify the contributions of the markers, leading to better accuracy. This also explains why the data-driven encoding works better for olygogenic traits, which depend on relatively few number of markers. For these olygogenic traits, a few markers are significantly associated with them. The data-driven encodings of these markers are close to the value of the traits and they can be more easily identified in the regression. Thus the prediction can be improved. On the contrary, for polygenic traits, which depend on a large number of markers, all these markers have similar associations. Thus the new encoding values are similar to each other. So the data-driven encoding is not able to improve the prediction performance much. In our future work, we would like to propose a theoretical analysis for the performance of the data-driven encoding.

## Results

As rrBLUP is one of the most commonly used methods for genetic trait prediction, in our experiments, we evaluate the prediction accuracy of rrBLUP for different encodings.

### Effects of different encodings

We first illustrate that different encodings lead to different prediction performances for epistasis model defined in Formula 2, thus a data-driven encoding has the benefit that we do not need to worry about the selection of the encodings. We use the Maize data set [7], which consists of two maize diversity panels with 300 Flint and 300 Dent lines developed for the European CornFed program. The two panels, Flint and Dent, were genotyped using a 50 k SNP array, which after removing SNPs with high rate of missing markers and high average heterozygosity, yielded 29,094 and 30,027 SNPs respectively. Both of them contain 261 samples. In this experiment, we use only Dent data set. For a pair of SNPs, we consider the following four combinations of encodings (*SNP*_1_, *SNP*_2_) as (*E*1 = {0, 1, 2}, {0, 1, 2}), (*E*2 = {0, 1, 2}, {2, 1, 0}), (*E*3 = {2, 1, 0}, {0, 1, 2}), (*E*4 = {2, 1, 0}, {2, 1, 0}).

We test all pairwise epistasis effects under the four combinations of encodings and rank the epistasis effects according to their mutual information to the trait. Then we measure the correlation of the ranks among these four combinations. Spearman's rank correlation is a popular method to compare two ranks. However, it requires that the two ranks have the same set of elements. While in our case, the ranks reported by different encodings overlap but may contain different SNP pairs. So we adopted an average accuracy correlation method [38]. The results for Dent trait Tass are shown in Table 1. We can see that the ranks from different encoding strategies have very low correlations, indicating that different encodings lead to completely different epistasis effects.

**Table 1:** The correlation between top MI (Mutual Information) ranks by different encoding.

Genotype Encoding	E1	E2	E3	E4
E1	1	0.003	0.001	0.001

E2	0.001	1	0.001	0.004

E3	0.001	0.001	1	0.001

E4	0.003	0.003	-	1

Next we compare the performance of rrBLUP for the two encodings {0, 1, 2} and {−1, 0, 1} and we apply rrBLUP on all the three traits of Dent under each encoding, respectively. We applied the epistasis model in Formula 2 and show the average results of 10-fold cross validation in Table 2. The performance is measured as the square of the person's correlation, or *r*^2^. The larger the *r*^2^, the better the regression is and the better the prediction is. As we can see, the two encodings lead to very different accuracies and {0, 1, 2} is a better encoding on the Dent data set.

**Table 2:** The *r*^2 ^of predicted trait value under encoding sets {0, 1, 2} and {−1, 0, 1} for the epistasis model in Formula 2 on the Dent data set.

Dataset	{0, 1, 2}	{−1, 0, 1}
Dent 1 Tass	0.59	0.457
Dent 2 DMC	0.562	0.481
Dent 3 DM Yield	0.321	0.211

### Simulated Data

We next compare the predictive performance of rrBLUP based on the traditional encoding {0, 1, 2} and the two versions of data-driven encodings we proposed. We simulate both polygenic traits and oligogenic traits. We randomly generate the genotype matrix *X *of size 100 × 500, namely 100 samples each with 500 genotypes. The trait is generated according to the formula $Y=\sum_{i=1}^{m}\beta_{i}X_{i}+e_{l}$. The coefficient of the markers *β_i _*and the residual error *e_l _*are also randomly generated. We set the coefficients of the first *s *genotypes, namely *β*_1_, *β*_2_,...,*β_s_*, as non-zero and of the remaining genotypes as 0. We vary the value of *s *as 5, 10, 20, 50, 100, with small *s *for oligogenic traits and large *s *for polygenic traits. Out of 100 samples, 90 are training samples and 10 are test sample. We randomly simulate 10 data sets and for each data set we conduct 10-fold cross validation. We compute the average prediction performance and show the results in Table 4.

**Table 3:** Performance of rrBLUP (average *r*^2^) on the traits of four real data sets under the traditional encoding vs. the hybrid data-driven encoding.

Data Set	Traditional Encoding	Hybrid Data-driven Encoding	Improvement
Rice: Pericarp.color	0.433	0.504	16.4%

Rice: Protein.content	0.176	0.177	0.6%

Pig: Trait 2	0.237	0.239	0.8%

Pig: Trait 4	0.203	0.218	7.4%

QTLMAS: Trait 1	0.358	0.361	0.8%

QTLMAS: Trait 2	0.187	0.18	-3.7%

Maize: Flint 1 TASS	0.47	0.492	4.7%

Maize: Flint 2 DMC	0.301	0.308	2.3%

Maize: Flint 3 DM_Yield	0.057	0.068	19.3%

Maize: Dent 1 Tass	0.59	0.616	4.4%

Maize: Dent 2 DMC	0.562	0.58	3.2%

Maize: Dent 3 DM_Yield	0.321	0.349	8.7%

**Table 4:** Performance (average *r*^2 ^over 10 randomly simulated data sets) of rrBLUP for different genotype encodings and different number of contributing genotypes *s*.

s	Traditional Encoding	Pure Data-driven Encoding	Hybrid Data-driven Encoding
5	0.1095	0.0239	**0.1334**

10	0.0569	0.0512	**0.0761**

20	0.1841	0.1334	**0.1882**

50	**0.1656**	0.0151	0.1108

100	**0.2494**	0.1420	0.2147

200	**0.2661**	0.1267	0.2073

We can see that when *s *is small, for example, *s *= 5, 10, 20, the traits are oligogenic and the performance of the hybrid encoding is better than that of the traditional encoding. However, when *s *is big, for example, *s *= 50, 100, 200, the traits are polygenic, the performance of the hybrid encoding is worse than that of the traditional encoding. We also observe that the pure data-driven encoding works poorly for all cases, indicating that it is important to also take into consideration the relationship of homozygous and heterozygous genotypes.

### Real data

Next we apply the new encoding strategy to four different data sets. As the pure data-driven encoding shows poor performance, we evaluate only the hybrid data-driven encoding. We compare the performance of rrBLUP on both the traditional encoding and the hybrid data-driven encoding and show the average *r*^2 ^of 10-fold cross validation.

The first data set is the Maize data set [7] which was used to evaluate the effects of different encodings in Section. As we can see in Table 3, for all six traits, the data-driven encoding achieves better performance than the traditional encoding does.

The second data set is the Asian rice, Oryza sativa, data set [39]. This data set was based on 44,100 SNP variants from 413 accessions of O. sativa, taken from 82 countries containing 34 phenotypes. We selected two phenotypes, one is polygenic (Protein.content), one is oligogenic (Pericarp.color). The data sets have 36,901 markers and 413 samples. We again vary the number of selected features as 500, 1000. As shown in Table 3, for the oligogenic trait (Pericarp.color), the performance of the data-driven encoding is significantly better than that of the traditional encoding. On the contrary, for the polygenic trait (Protein.content), the two encodings achieve similar performance. This is consistent with our simulation, namely the data-driven encoding works better for oligogenic traits.

The third data set is Pig data set, which is a collection data on male and female pigs born since 2000 and was taken from [5] and consists of 3,534 animals from a single PIC nucleus pig line yielding 52,842 SNPs with five measured traits (phenotypes). Only traits 2 and 4 were selected for study here. As described in [5], genotypes were sequenced from the Illumina PorcineSNP60 chip and full pedigree information is available, which we did not use in this study. In the original study, trait 2 was rescaled by a weighted mean of corrected progeny phenotypes. Whereas trait 4 was corrected for environmental factors such as year of birth and location. Genotypes were filtered for minor allele frequency less than 0.001 and with missing genotypes less than 10%. The original study used AlphaImpute to impute any missing data [14]. As we can see in Table 3, for both traits, the data-driven encoding achieves better performance.

The fourth data set is QTLMAS data set, which was taken from the QTL-MAS Workshop, which was held on May 17-18, 2010 in Poznan Poland [1]. The data set consists of 3,226 individuals over five generations (F0-F4) with 20 founders, five male and 15 females. There were two phenotype traits, the first a quantitative trait and the second a binary trait. Only the first four generations (2,326 individuals) have phenotype records. The genome is approximately 500 million bp with five chromosomes, each 100 million bp. In total, each individual was genotypes for 10,031 biallelic SNPs. We can see in Table 3, data-driven encoding achieves better performance for Trait 1 and worse performance for Trait 2.

For genetic prediction, an improvement of 5% is considered as significant. As shown in Table 3, in general the data-driven encoding is able to improve the prediction performance and in many cases the improvement is significant. Thus even for single marker model, the data-driven encoding is superior to the traditional encoding.

We also applied the data-driven encoding strategy on the epistasis model shown in Formula 2. We computed all pairs of epistasis effects and selected the top 2,000 pairs, using their relevance, measured by mutual information, to the trait. We then include these top 2,000 pairs of epistasis effects in Formula 2. We conducted the experiments only on the Maize data set as it is the only data set that is small enough for an extensive search.

As we can see in Table 5, the performance of epistasis is better for most of the traits when using the data-driven encoding. For the two traits where the traditional encoding is better, the performance of the data-driven encoding is only slightly worse than that of the original encoding.

**Table 5:** Performance (average *r*^2^) of rrBLUP for the single marker model (Formula 1) and epistasis model (Formula 2) under the traditional encoding vs. the data-driven encoding.

Dent				
**Phenotype**	**rrBLUP (T)**	**rrBLUP (D)**	**Epistasis (T)**	**Epistasis (D)**

1	0.590	0.616	0.590	**0.616**
2	0.552	0.58	0.552	**0.58**
3	0.321	0.349	**0.356**	0.349

**Flint**				

Phenotype	rrBLUP (T)	rrBLUP (D)	Epistasis (T)	Epistasis (D)

1	0.470	0.492	0.476	**0.493**
2	0.301	0.308	**0.316**	0.312
3	0.057	0.068	0.096	**0.102**

rrBLUP (T) is the single marker model under the traditional encoding. rrBLUP (D) is the single marker model under the data-driven encoding. Epistasis (T) is the epistasis model under the traditional encoding. Epistasis (D) is the epistasis model under the data-driven encoding.

## Conclusions

In this work, we showed that the genetic trait prediction problem heavily depends on the encoding of genotypes, especially when epistasis effects are considered. We developed a data-driven encoding method which is simple but effective with the benefits that we don't need to choose between different encodings. Out experiments show that the data-driven encoding is able to improve the prediction accuracy for both single marker model and epistasis model, especially for olygogenic traits. To our knowledge, this is the first work that discusses the effects of encodings for genetic trait prediction problem. In our future work, we would like to theoretically analyze the effects of the data-driven encoding for the genetic trait prediction problem.

## Competing interests

The authors declare that they have no competing interests.

## Authors' contributions

LP proposed the study. ZYW carried out the experiments to demonstrate the effect of encoding, DH designed and implemented the algorithms, and carried out the comparison experiments. All the authors read and approved the manuscript.
